# Chondroitin Sulfate *N*-acetylgalactosaminyltransferase-2 Contributes to the Replication of Infectious Bursal Disease Virus via Interaction with the Capsid Protein VP2

**DOI:** 10.3390/v7031474

**Published:** 2015-03-23

**Authors:** Lizhou Zhang, Xiangang Ren, Yuming Chen, Yulong Gao, Nian Wang, Zhen Lu, Li Gao, Liting Qin, Yongqiang Wang, Honglei Gao, Kai Li, Lili Jiang, Hongyu Cui, Changjun Liu, Yanping Zhang, Xiaole Qi, Xiaomei Wang

**Affiliations:** 1 Division of Avian Infectious Diseases, State Key Laboratory of Veterinary Biotechnology, Harbin Veterinary Research Institute, the Chinese Academy of Agricultural Sciences, Harbin 150001, China; E-Mails: zhanglizhou88@126.com (L.Z.); renxiangang@caas.cn (X.R.); cym27@sina.cn (Y.C.); ylg@hvri.ac.cn (Y.G.); wnian@outlook.com (N.W.); luzhen9122@163.com (Z.L.); gaoli0820@163.com (L.G.); qinlt2013@163.com (L.Q.); yqw@hvri.ac.cn (Y.W.); ghl@hvri.ac.cn (H.G.); likaihvri@163.com (K.L.); lyjllfxy1024@163.com (L.J.); cuihongyu@caas.cn (H.C.); liucj93711@hvri.ac.cn (C.L.); zhangyanping03@caas.cn (Y.Z.); 2 Jiangsu Co-Innovation Center for Prevention and Control of Important Animal Infectious Disease and Zoonoses, Yangzhou 225009, China

**Keywords:** CSGalNAcT2, Golgi apparatus, infectious bursal disease virus, growth, VP2

## Abstract

Infectious bursal disease virus (IBDV) is a birnavirus that causes a highly contagious immunosuppressive disease in young chickens. The capsid protein VP2 of IBDV plays multiple roles in its life cycle. To more comprehensively understand the functions of VP2 involved in the communication between virus and host, we used yeast two-hybrid screening to identify the cellular factors that interact with this protein. We found that chondroitin sulfate *N*-acetylgalactosaminyltransferase-2 (CSGalNAcT2), a typical type II transmembrane protein located in Golgi apparatus, could interact with VP2, and we confirmed this interaction by co-immunoprecipitation and confocal laser scanning microscopy assays. Additionally, up-regulation of CSGalNAcT2 during IBDV infection was observed. Overexpression and siRNA-mediated knockdown of CSGalNAcT2 assays suggested that CSGalNAcT2 promoted IBDV replication. Moreover, this enhancing effect of CSGalNAcT2 could be inhibited by Brefeldin A, which is a Golgi-disturbing agent. This indicated that the integrity of Golgi apparatus structure was involved in the function of CSGalNAcT2. Taken together, we concluded that CSGalNAcT2, located in the Golgi apparatus, contributed to the replication of IBDV via interaction with VP2.

## 1. Introduction

Infectious bursal disease (IBD, also called Gumboro disease) is an acute, highly contagious immunosuppressive disease of young chickens [1,2]. The etiological agent, infectious bursal disease virus (IBDV), leads to destruction of the central immune organ of the chicken (bursa of Fabricius), leading to immunosuppression and reduced response to vaccines, which in turn increases susceptibility to other pathogens [3]. IBD has become one of the major problems threatening the global commercial poultry industry [4,5,6].

IBDV belongs to the genus *Avibirnavirus*, of the family *Birnaviridae* [7,8]. Members of this family are characterized by a double-stranded RNA (dsRNA) genome consisting of two segments (A and B) [3]. Segment A (about 3.2 kb) has two partially overlapping open reading frames (ORFs). The larger ORF encodes a 110-kDa polyprotein precursor in the order NH2−pVP2−VP4−VP3−COOH. The polyprotein precursor is cotranslationally processed by the viral protease VP4 into pVP2 (48 kDa), VP3 (32 kDa), and VP4 (28 kDa) [9,10,11]. pVP2 is further processed by several proteolytic cleavages at its C terminus, converting it into mature VP2 (40 kDa), during virus assembly [12,13]. The small ORF encodes VP5, a 17-kDa nonstructural protein, which is dispensable for viral replication [14,15]. Segment B (about 2.8 kb) encodes a single viral protein, VP1 (91 kDa), an RNA-dependent RNA polymerase.

VP2 is the only component of the icosahedral IBDV capsid [16,17], which plays multiple roles in the virus life cycle, including antigenic variation [18,19], virulence [3,20,21], as well as cell tropism [22,23,24]. However, little is known about the intracellular interaction between VP2, an important multifunctional protein, and host cells. 

Viruses are molecular machines comprising viral and occasionally cellular macromolecules [25]. When a virus enters a cell, the subsequent steps in its replication cycle involve interactions between different types of viral components and much more complex pools of host factors. Many viruses not only evade host defenses, but also take advantage of interactions with host proteins to ensure replication. Only a few factors, including the glucocorticoid-induced leucine zipper protein [26], regulatory subunit p85α of PI3K [27], and voltage-dependent anion channel 2 (VDAC2) [28], have been reported to play different roles during the IBDV infection. So, it is necessary to understand more about the interaction between host cells and IBDV.

In this study, we firstly identified that CSGalNAcT2, a Golgi protein, promoted IBDV infection via interaction with the viral capsid protein VP2, yielding novel insights into the pathogenic mechanism of IBDV.

## 2. Materials and Methods

### 2.1. Cells and Virus

DF-1 (immortalized chicken embryo fibroblast) cells were cultured in Dulbecco’s modified Eagle’s medium (DMEM; Invitrogen, Carlsbad, CA, USA) supplemented with 10% fetal bovine serum (FBS; HyClone Laboratories Inc., South Logan, UT, USA) at 37 °C in a 5% CO_2_ incubator. Chicken embryo fibroblast (CEF) cells were prepared from 10-day-old specific-pathogen-free (SPF) chicken embryos. IBDV Gt strain, which is a cell-culture adapted strain, was attenuated and identified in our laboratory as described previously [29]. 

### 2.2. Plasmids

The eukaryotic expression vectors pCAGGS-Flag or pCAGGS-HA, including a Flag-tag or HA-tag, were reconstructed from pCAGGS [30] and used for construction of the following plasmids. The pGtVP2 plasmid encoded the VP2 protein of IBDV Gt strain fused to an HA-tag at its *N*-terminus. The CSGalNAcT2 gene was cloned from DF-1 cells using primers shown in Table 1, designed according to the sequence in GenBank (XM_004941969.1). The pCSGalNAcT2 plasmid encoded the CSGalNAcT2 protein (62 kDa) fused to a Flag-tag at its *C*-terminus. p∆CSGalNAcT2 encoded the truncated CSGalNAcT2 mutant (58 kDa), of which the signal peptide and transmembrane region (amino acids 1–37 at the *N*-terminal) was deleted. p∆CSGalNAcT2 was constructed only for use in the co-immunoprecipitation assay, while pCSGalNAcT2 was used for other experiments.

**Table 1:** Primers used in this study.

Primers	Sequences(5'-3')	Usage	Products Size
GtVP2-U	CC*GAATTC*ATGACAAACCTGCAAGAT	Construction for pGtVP2	1335 bp
GtVP2-L	CC*GGTACC*TTATGCTCCTGCAATCTTCAG		
Bait-VP2-U	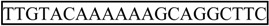 ATGACAAACCTGCAAGAT	Construction for pDONR221-GtVP2	1365 bp
Bait-VP2-L	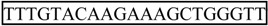 TTATGCTCCTGCAATCTTCAG		
Gal -U	CA*GAATTC*ATGAGAATGCCCAGAAGAGGCT	Construction for pCSGalNAcT2	1635 bp
Gal -L	AC*GGTACC*ATTAACCCACTGTTTCGCTGTT		
∆Gal -U	CA*GAATTC*ATGCCACAGACAGATAGTAAT	Construction for p∆CSGalNAcT2	1524 bp
∆Gal -L	AC*GGTACC*ATTAACCCACTGTTTCGCTGTT		
β-actin-U	CTGTGCCCATCTATGAAGGCTA	Quantification for β-actin	138 bp
β-actin-L	ATTTCTCTCTCGGCTGTGGTG		
Gal-F	AAGAGCCCCAAGGTCAATGG	Quantification for CSGalNAcT2	138 bp
Gal-R	GACGCCGTATTCACTAGGCA		

The attB sequences used for recombinant reaction with pDONR221 vector are shown in box and the introduced restriction enzyme sites are marked in italics.

### 2.3. Virus Infection and Titration

DF1 cells that were 80%–90% confluent in 6-well or 12-well plates were infected with IBDV Gt strain at an MOI of 0.01. After incubation for 1 h at 37 °C, DF-1 cells were washed three times with phosphate-buffered saline (PBS, pH 7.4) and were then cultured in fresh medium. At 24 h or 48 h post-infection, the cell supernatants were collected to detect viral infectivity titres by infecting the CEF cells and the titres were calculated as TCID_50_ per 100 μL using the Reed–Muench formula [31]. The DF1 cell monolayers were also washed and lysed immediately using the western and immunoprecipitation lysis buffer (Beyotime Institute of Biotechnology, Beijing, China) to detect VP2 or pVP2 expression by Western blotting.

### 2.4. Viral RNA Isolation and Real-Time RT-PCR Analysis

To determine the genomic RNA copies of IBDV, the viral RNA was extracted from cell supernatants with the Viral RNA kit (Omega Biotek, Norcross, GA, USA) and then reverse transcribed into cDNAs with M-MLV reverse transcriptase (Invitrogen). The IBDV genome copy numbers were quantified by quantitative real-time PCR as described previously [32].

### 2.5. Yeast Two-Hybrid Screening

The ProQuest Two-Hybrid System (Invitrogen) was used to screen the host proteins interacting with VP2 of IBDV. Briefly, the cDNA library of bursa of Fabricius cells from the 3-week SPF chicken was constructed according to the supplier’s protocol. The VP2 gene of the IBDV Gt strain was cloned using attB-modified custom primers (Table 1) and the PCR products recombined with the pDONR221 vector, yielding the entry clone pDONR221-GtVP2, which was then recombined with the pDEST32 vector (containing the GAL4 DNA-binding domain) to form the bait plasmid pDEST32-GtVP2. Self-activation of the bait protein was detected by cotransforming pDEST32-GtVP2 and pDEST22 (containing the GAL4 DNA-activation domain) into the yeast strain MaV203. When no self-activation was detected, the cDNA library and the bait plasmids were introduced into the yeast strain Mav203 and selected on SD/-Leu/-Trp plates. Clones containing interacting proteins were identified on SD/-Leu/-Trp/-His and SD/-Leu/-Trp/-Ura plates, as well as by X-gal assay. Positive control (Krev1 and RalGDS-wt) and negative control (Krev1 and RalGDS-m2) were used as indicated by the manufacturer (Invitrogen).

### 2.6. Analysis of Differentially Expressed Genes

The total RNA of CEF cells infected with the IBDV Gt strain at an MOI of 0.01 was isolated with RNAiso Plus (D9108B, TaKaRa, Shiga, Japan) at 24 h post-infection and treated with DNase I to remove potential genomic DNA contamination. Uninfected CEF cells were used as control. Then, the RNAs were subjected to differential gene expression analysis at the Beijing Genomics Institute (Shenzhen, China). In brief, the libraries were constructed from the total RNA, after mRNA enrichment, fragment interruption, addition of adaptors, size selection, and PCR amplification, and were then sequenced by IlluminaHiSeqTM. To obtain clean reads, raw reads were filtered by discarding bad sequences (adaptor reads, N10% unknown bases, and low quality reads). The number of clean tags exclusively mapped for each gene was counted and normalized using the reads-per-kb-million-reads method [33]. In this study, a false discovery rate (FDR) was applied to determine the threshold *p*-value. We used FDR < 0.001 and an absolute value of log_2_ ratio (IBDV/Mock) > 1 to identify significantly different differentially expressed genes as previously described [34].

### 2.7. Validation of CSGalNAcT2 Gene Expression

To confirm the results of differential gene expression analysis, the same RNAs were reverse transcribed into cDNAs using M-MLV reverse transcriptase (Invitrogen) and used for the relative quantification of the CSGalNAcT2 gene by real-time RT-PCR, using SYBR Green Realtime PCR Master Mix (Toyobo, Osaka, Japan). CSGalNAcT2 gene expression was normalized to that of the beta-actin gene. The primers used for real-time RT-PCR are shown in Table 1.

### 2.8. Co-Immunoprecipitation and Western Blot Analysis

DF1 cells were seeded into 6-well plates and cultured in monolayers to 80%–90% confluence prior to co-transfection with 2 μg p∆CSGalNAcT2 and 2 μg pGtVP2 or 2 μg empty vectors as controls, using Lipofectamine 2000 (Invitrogen). At 36 h post-transfection, the transfected cells were washed three times with cold PBS and the cell lysates were prepared by adding 200 μL western and immunoprecipitation lysis buffer (Beyotime Institute of Biotechnology, Beijing, China) containing 1 mM PMSF (Beyotime Institute of Biotechnology). The clarified lysates were precleared with protein A/G beads (Santa Cruz, Dallas, TX, USA); after centrifugation, 40 μL supernatant was used as the input, and another 160 μL supernatant was incubated with the anti-VP2 monoclonal antibody (mAb) [35] for 2 h at 4 °C, before adding protein A/G beads for overnight at 4 °C. Finally, the beads complexes were washed three times with cold PBS, boiled in 5 × sample loading buffer for 10 min, and subjected to electrophoresis on 12% SDS-PAGE gels, followed by immunoblot analysis with either the anti-FLAG (Sigma, St Louis, MO, USA) or the anti-VP2 mAbs [35], and then IRDye^®^ 800CW goat anti-mouse IgG (LiCor BioSciences, Lincoln, NA, USA). Blots were detected using an Odyssey Infrared Imaging System (LiCor BioSciences, Lincoln, NA, USA).

### 2.9. Confocal Laser Scanning Microscopy Assay

For confocal laser scanning microscopy assays, DF1 cells grown in glass-bottomed culture dishes (NEST Biotechnology Co. LTD, Wuxi, China) were co-transfected with pCSGalNAcT2 and pGtVP2 as described above. Single transfections with 4 μg pCSGalNAcT2 or 4 μg pGtVP2 were performed as controls. At 36 h post-transfection, the cells were fixed with 4% paraformaldehyde, permeabilized with 0.1% Triton X-100, and blocked with 3% bovine serum albumin (BSA). Then, cells were incubated with the anti-FLAG monoclonal antibody and the anti-HA polyclonal antibody (Sigma) followed by addition of the corresponding FITC- or TRITC-conjugated secondary antibody for 1 h at room temperature. After washing three times with PBST containing 0.05% Tween-20, cells were stained with DAPI (Beyotime Institute of Biotechnology) for 10 min and analyzed using a Leica SP2 Confocal system (Leica Laser Technik, Heidelberg, Germany). Additionally, to determine whether the exogenous CSGalNAcT2 was expressed and located in the Golgi apparatus, we used confocal microscopy to evaluate the location of the exogenous CSGalNAcT2 and the endogenous GOLGA2 protein, a known marker for the Golgi apparatus, using GOLGA2 antibody (Sigma).

### 2.10. CSGalNAcT2 Overexpression Assay

To investigate whether CSGalNAcT2 has an effect on IBDV replication, the CSGalNAcT2 protein was overexpressed in DF1 cells by transfecting 4 μg pCSGalNAcT2 plasmids to the cells in 6-well plates. At 36 h post-transfection, cells were infected with the IBDV Gt strain at an MOI of 0.01. At 48 h post-infection, the culture supernatants and cell monolayers were harvested for viral titration (TCID_50_) and VP2 expression detection (by Western blotting) as described above.

### 2.11. RNA Interference of CSGalNAcT2

Small interference RNAs were synthesized by Shanghai GenePharma Co., Ltd. (Shanghai, China) and used for the interference assay of CSGalNAcT2. The sequences of siRNA for targeting CSGalNAcT2 (GenBank XM_004941969.1) are shown in Table 2. The efficiency of knockdown by the synthesized siRNA was evaluated with both exogenous and endogenous CSGalNAcT2. For the exogenous CSGalNAcT2, 2 μg pCSGalNAcT2 was co-transfected with 100 pmol of the siRNA into DF1 cells cultured in 12-well, using Lipofectamine 2000. After 48 h, the expression of CSGalNAcT2 was detected by Western blotting using the anti-Flag antibody. For the endogenous CSGalNAcT2, DF1 cells were seeded into 12-well plates and cultured in monolayers to 50% confluence. Then, cells were transfected with 60 pmol siRNA per well. The expression of endogenous CSGalNAacT2 was detected using real-time RT-PCR at 48 h post-transfection.

**Table 2:** The sequences of siRNA targeting CSGalNAcT2 gene.

Name	Sequences (5'-3')	Orientation	Position (bp)
**1# siRNA**	CCAAAGAGCAAGCAUCCAATT	sense	341–359
	UUGGAUGCUUGCUCUUUGGTT	antisense	341–359
**2# siRNA**	GCGAGGUCCUGAUGUUCUUTT	sense	1070–1088
	AAGAACAUCAGGACCUCGCTT	antisense	1070–1088
**3# siRNA**	GCAUGUGUAUCCAGUCCAATT	sense	1493–1511
	UUGGACUGGAUACACAUGCTT	antisense	1493–1511
**NC siRNA**	UUCUCCGAACGUGUCACGUTT	sense	no
	ACGUGACACGUUCGGAGAATT	antisense	no

NC stands for negative control.

### 2.12. Brefeldin A Assay

To investigate whether the effect of CSGalNAcT2 on IBDV is associated with the Golgi apparatus, Brefeldin A (Beyotime Institute of Biotechnology) [36] was used to disrupt the Golgi apparatus. Firstly, to confirm the effect of Brefeldin A on the Golgi apparatus and the location of exogenously expressed CSGalNAcT2, DF1 cells transfected with pCSGalNAcT2 were treated with Brefeldin A, after which the cells were fixed and permeabilized for immunofluorescence assay. Next, cells were divided into four groups to investigate the effect of Brefeldin A on the promotion of IBDV replication by CSGalNAcT2. Groups I and II were transfected with 4 μg of the empty plasmid pCAGGS, and groups III and IV were transfected with 4 μg pCSGalNAcT2. Then, DF1 cells in all groups were incubated with the IBDV Gt strain at an MOI of 0.01, for 1 h at 37 °C. After washing the cells three times with PBS, groups I and III were supplied with fresh medium containing 1 μM Brefeldin A for another 48 h, while groups II and IV received DMSO, the solvent of Brefeldin A, as control. After 48 h, VP2 or pVP2 expression (by Western blotting) and viral genome copy numbers (by quantitative real-time RT-PCR) were analyzed.

### 2.13. Statistical Analysis

Statistical analyses were performed using GraphPad Prism 6.0 software (GraphPad Software, San Diego, CA, USA). Student’s *t*-test and one-way ANOVA was used to compare viral titres and genomic RNA copies. A *p*-value of <0.05 was considered significantly different.

## 3. Results

### 3.1. Host Cell Proteins Interact with VP2 of IBDV

**Figure 1:**
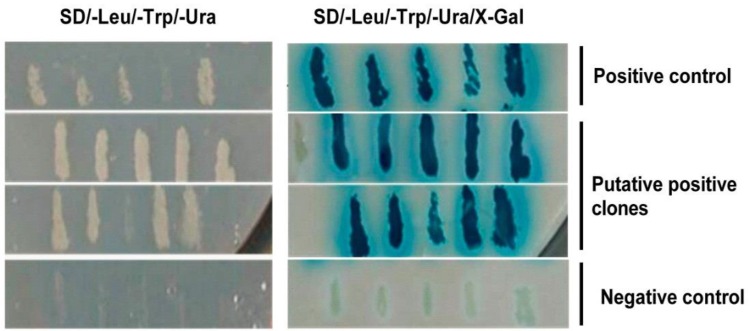
Screening of host cell proteins interacting with VP2 of IBDV by yeast two-hybridization assay. Briefly, the capsid protein VP2 of the IBDV Gt strain was used as the bait in the ProQuest yeast two-hybrid system to screen a cDNA library generated from the chicken bursa of Fabricius cells. The interactions were examined by SD/-Leu/-Trp/-Ura plates and X-gal assay. Ten putative positive colonies were tested for β-galactosidase activity in two additional rounds of selection. The positive (Krev1 and RalGDS-wt) and the negative controls (Krev1 and RalGDS-m2) were performed during each selection round.

To identify the host cellular proteins interacting with the capsid protein VP2 of IBDV, we used VP2 of the Gt IBDV strain as bait in a yeast two-hybrid system, to screen a cDNA library with a titre of 1.2 × 10^6^ CFU, generated from chicken bursa of Fabricius cells. In the initial screen, 35 hits were obtained by X-Gal assay on SD/-Leu/-Trp selection plates. In the second round of selection, 10 putative positive clones were identified on SD/-Leu/-Trp/-Ura plates and these were subsequently tested for β-galactosidase activity (Figure 1). The plasmids from 10 clones were extracted and sequenced. Among these clones, six clones were found to be in frame with GAL4 DNA-activation domain, while the others were false positives. These six positive clones were assessed by Basic Local Alignment Search Tool (BLAST) analysis in NCBI. Two clones represented Gallus mitochondrial DNA (AP003321.1); the remainder represented tumor protein p53-binding protein (XM_413957.2), O-linked *N*-acetylglucosamine transferase (XM_001232518.1), chondroitin sulfate *N*-acetylgalactosaminyltransferase-2 (CSGalNAcT-2; XM_004941969.1), and stathmin (X65458.1), respectively.

### 3.2. Expression of the Gene Encoding CSGalNAcT2 is Up-Regulated during IBDV Infection

**Figure 2:**
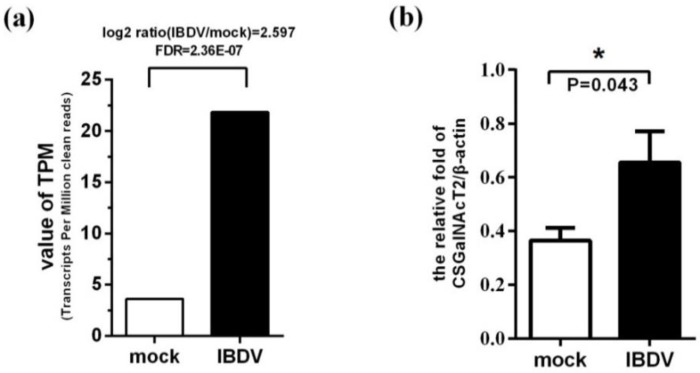
CSGalNAcT2 is up-regulated during IBDV infection. (**a**) Differential gene expression analysis by gene chip assay. The total RNA of CEF cells infected by IBDV Gt strain at 24 h post-infection was extracted for gene chip analysis. The value of transcripts per million clean reads (TPM) of CSGalNAcT2 showed that CSGalNAcT2 was up-regulated after IBDV infection. We set the false discovery rates (FDR) < 0.001 and | log_2_ ration (IBDV/mock) | > 1 as significantly different differentially expressed genes. (**b**) Detection of CSGalNAcT2 expression by real-time RT-PCR. Real-time RT-PCR was performed to detect the CSGalNAcT2 expression of CEF cells at 24 h post-infection with IBDV Gt strain. Levels of CSGalNAcT2 were normalized to that of beta-actin, which further confirmed the results of gene chip analysis. The results are representative of three independent experiments. Values are the mean ± SEM from three triplicate wells. *, *p* < 0.05 means significantly different.

To identify the cellular genes associated with IBDV infection, genes differentially expressed in CEF cells after infection with IBDV were analyzed by gene chip assay. Transcription of the CSGalNAcT2-encoding gene was up-regulated in the experimental group compared with the mock group (Figure 2a). Real-time RT-PCR was performed to detect CSGalNAcT2 gene expression of infected or uninfected CEF cells, and confirmed that CSGalNAcT2 gene expression was up-regulated during IBDV infection (Figure 2b).

### 3.3. VP2 of IBDV Interacts with Host Cell Protein CSGalNAcT2 in the Golgi Apparatus

**Figure 3:**
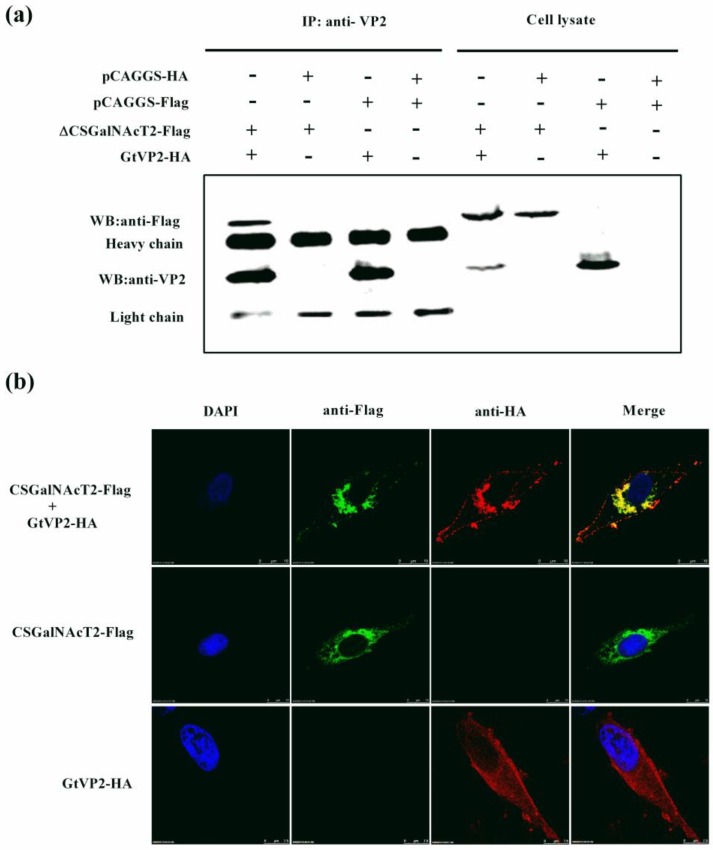

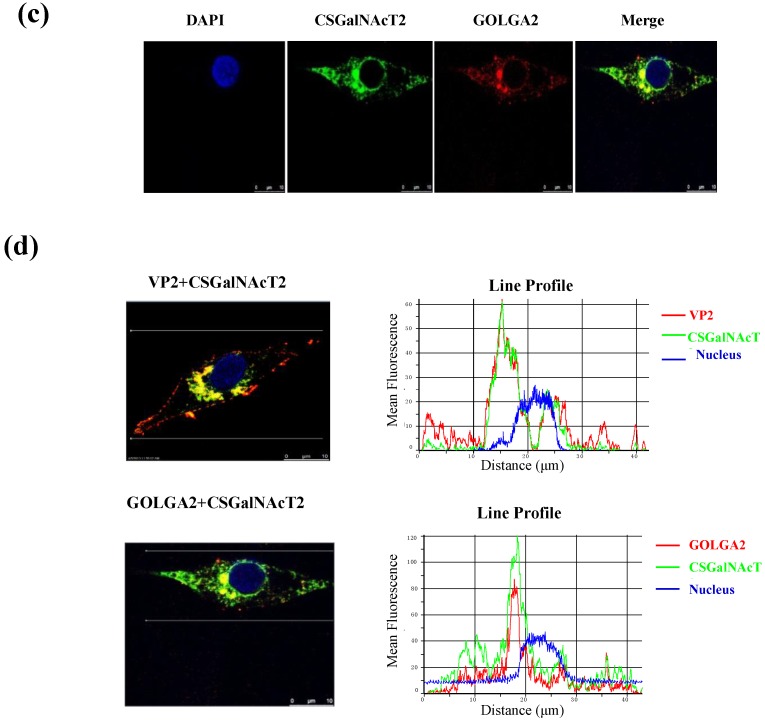
Identification of the interaction of IBDV VP2 and CSGalNAcT2. (**a**) Co-immunoprecipitation of IBDV (Gt) VP2 and exogenous ∆CSGalNAcT2-Flag in DF-1 cells. DF-1 cells were transfected with 4 μg of the indicated plasmids. At 36 h post-transfection, the cell lysates were prepared and immunoprecipitated (IP) with the anti-VP2 monoclonal antibody. VP2 (40 kDa) and ∆CSGalNAcT2-Flag (59 kDa) in the immune complex were detected with the mouse anti-VP2 or anti-Flag antibodies. The light and heavy chains were the component of the anti-Flag antibody used for immunoprecipitation. (**b**) Co-localization of IBDV (Gt) VP2 and CSGalNAcT2. DF1 cells were co-transfected with pCSGalNAcT2 and pGtVP2. Cells were fixed at 36 h post-transfection and subjected to indirect immunofluorescence assay to detect CSGalNAcT2 (green) and VP2 (red), using the mouse anti-Flag and rabbit anti-HA antibodies. The position of the nucleus was indicated by DAPI (blue) staining. (**c**) Exogenous CSGalNAcT2 was expressed and located in the Golgi apparatus, which was marked by the GOLGA2, a Golgi marker. (**d**) Quantitative analysis of the correlation between CSGalNAcT2 and VP2 or GOLGA2, using Image-Pro Plus 6.0 software. The consistent trends between red and green curves indicated that co-localization resulted from a strong correlation in expression.

To further confirm the interaction between VP2 and CSGalNAcT2, co-immunoprecipitation assay were performed by transiently co-expressing ∆CSGalNAcT2 (lacking amino acids 1–37) and VP2 in DF1 cells. When the lysate of cells expressing ∆CSGalNAcT2 and VP2 were immunoprecipitated with an anti-VP2 monoclonal antibody, the Flag-tagged ∆CSGalNAcT2 was detected with the anti-flag antibody (Figure 3a), indicating that VP2 can interact with CSGalNAcT2. 

Furthermore, confocal microscopy assay was performed to determine the subcellular location of VP2 and CSGalNAcT2. DF1 cells were transfected to express VP2 and CSGalNAcT2; the corresponding antibodies were added for immunostaining and cells were observed by microscopy. When independently expressed, VP2 was distributed throughout the cytoplasm, while CSGalNAcT2 was close to the cell nucleus. Interestingly, the location of VP2 was markedly altered upon co-expression with CSGalNAcT2. The typical cytoplasmic distribution of VP2 was altered to an accumulation around the cell nucleus (Figure 3b). 

This location of CSGalNAcT2 was narrowed down to the Golgi apparatus, because CSGalNAcT2 also co-localized with GOLGA2, a known marker protein for the Golgi apparatus (Figure 3c). Moreover, to quantitatively analyze the correlation between CSGalNAcT2 and VP2 or GOLGA2, Image-Pro Plus 6.0 software (Media Cybernetics Inc., Bethesda, MD, USA) was used to calculate the mean fluorescence intensity of the line profile of the co-localization image. As shown in Figure 3d, the red and green curves showed consistent trends, indicating that the co-localization was resulted from the strong correlation between CSGalNAcT2 and VP2 or GOLGA2. Taken together, CSGalNAcT2 interacts with VP2 in the Golgi apparatus.

### 3.4. Overexpression of CSGalNAcT2 Promotes IBDV Replication

**Figure 4:**
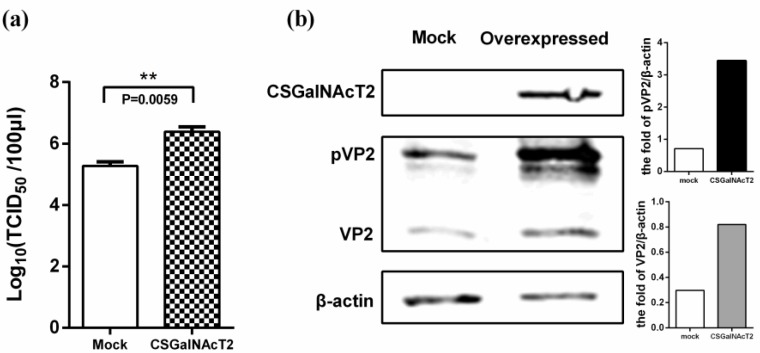
Overexpression of CSGalNAcT2 promotes IBDV replication. To determine the effect of up-regulation of CSGalNAcT2, overexpression assays were performed. DF1 cells were transfected with 4 μg pCSGalNAcT2 in 6-well plates. Then, the cells were infected with IBDV Gt strain at 36 h post-transfection. At 48 h post-infection, the cell supernatants were collected for determining the viral titre and the cell monolayers were lysed for detecting the VP2 expression levels. (**a**) The viral titre in the supernatant was determined by TCID_50_. The titre of the overexpression group was markedly higher than that in the mock. (**b**) The intracellular viral load was reflected by the VP2 or pVP2 expression levels, as determined using Western blotting. After normalized to beta-actin, the VP2 or pVP2 content in the overexpression group presented three to five times higher than that in the mock group. Values represent the mean ± SEM of three independent experiments. **, *p* < 0.01, significantly different.

To investigate the effect of up-regulation of CSGalNAcT2 on IBDV replication, DF1 cells were infected with IBDV at an MOI of 0.01, after transfection with pCSGalNAcT2, and the viral titre in the supernatant was determined by TCID_50_ at 48 h post-infection. The viral titre in the CSGalNAcT2 overexpression group was markedly higher than that in the mock group (Figure 4a). Moreover, both the amount of pVP2 and mature VP2 protein in the cells increased (Figure 4b), which was consistent with the viral titre in the supernatant.

### 3.5. Knockdown of CSGalNAcT2 Inhibits IBDV Replication

**Figure 5:**
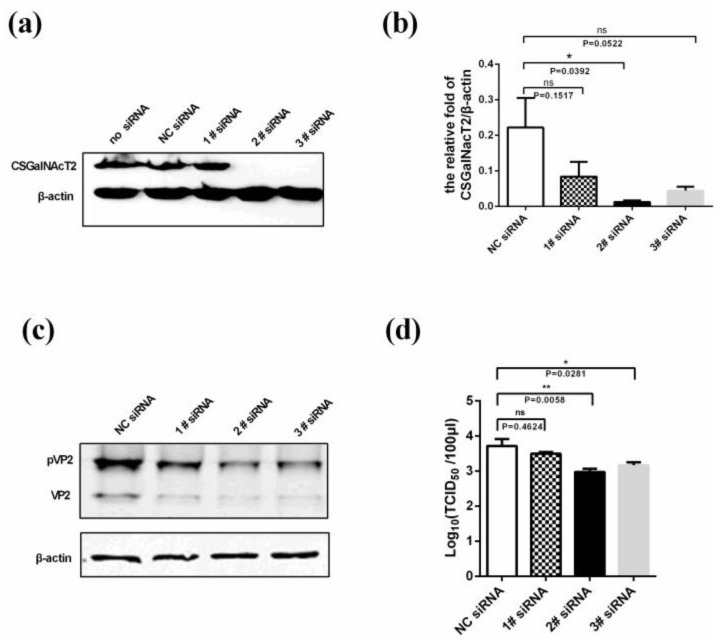
Knockdown of CSGalNAcT2 inhibits IBDV growth. (**a**) Interference efficiency of siRNAs against exogenous CSGalNAcT2. In DF1 cells, 2 μg pCSGalNAcT2 was co-transfected with 100 pmol negative control siRNA (NC), 1# siRNA, 2# siRNA, or 3# siRNA. Then, CSGalNAcT2 expression was detected using anti-Flag antibody; the results showed that the 2# siRNA and 3# siRNA had obvious effects. (**b**) The interference efficiency of these siRNAs against endogenous CSGalNAcT2. DF1 cells grown in 12-well plates were transfected with 60 pmol siRNA, and the interference efficiency was determined by real-time RT-PCR at 48 h post-transfection. The results further confirmed the superior interference efficiency of the 2# siRNA and 3# siRNA. Subsequently, cells were infected with the IBDV Gt strain at 48 h post-knockdown of endogenous CSGalNAcT2. IBDV in the cells (**c**) and supernatants (**d**) were determined by detecting the content of VP2 or pVP2 protein and viral titre at 24 h post-infection. Knockdown of CSGalNAcT2 inhibited IBDV growth. Values represent the mean ± SEM of three independent experiments. *, *p* < 0.05, significantly different; **, *p* < 0.01; ns, no significant difference.

To further confirm the role of CSGalNAcT2 in the IBDV life cycle, specific siRNAs were synthesized to target CSGalNAcT2 in DF1 cells. Firstly, we detected the efficiency of these siRNAs against exogenous CSGalNAcT2, and found that 2# and 3# siRNA efficiently interfered with the expression of CSGalNAcT2, but that 1# siRNA was markedly less efficient (Figure 5a). Then, the 2# and 3# siRNA were used to knock down the endogenous CSGalNAcT2 expression in DF1 cells, of which the interference efficiency of endogenous CSGalNAcT2 expression was confirmed by real-time RT-PCR (Figure 5b). At 48 h post-transfection, these cells were infected with IBDV at an MOI of 0.01; the viral titre and the viral protein expression levels were detected at 24 h post-infection. Compared with the mock group (NC siRNA) and the 1# siRNA, the 2# and 3# siRNA had a more markedly inhibitory effect on IBDV pVP2 or VP2 expression (Figure 5c,d). Furthermore, inhibition of viral protein expression correlated positively with the interference efficiency of siRNAs.

### 3.6. The Integrity of the Golgi Apparatus is Involved in the CSGalNAcT2 Effect on IBDV Replication

**Figure 6:**
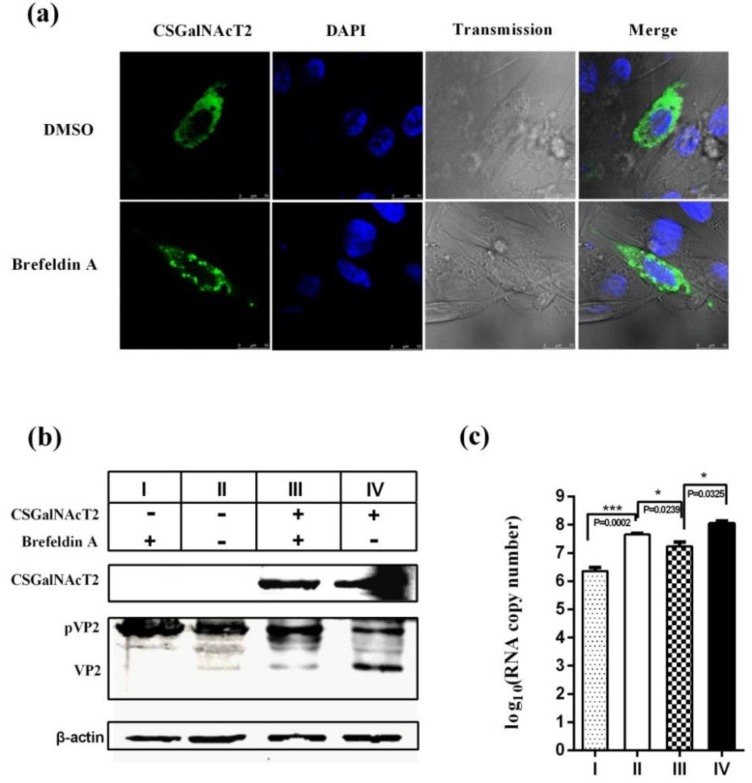
The integrity of the Golgi apparatus is involved in the effect of CSGalNAcT2 on IBDV replication. (**a**) To confirm the effect of Brefeldin A on the Golgi apparatus and the location of the exogenous CSGalNAcT2, DF1 cells transfected with CSGalNAcT2 were treated with Brefeldin A or DMSO for 48 h. Then, cells were fixed and permeabilized for immunofluorescence assay. The location of CSGalNAcT2 is indicated by FITC (green) and the whole cellular morphology is shown under transmission conditions. DAPI (blue) stains the cell nucleus. The exogenously expressed CSGalNAcT2 was dispersed cytoplasmically, rather than perinuclear, after Brefeldin A treatment, which suggested that Brefeldin A dispersed the structure of the Golgi apparatus and the exogenous CSGalNAcT2 was expressed and located in this organelle. Then, cells were divided into four groups to evaluate the effect of Brefeldin A on the enhancement of IBDV replication by CSGalNAcT2. The intracellular VP2 levels (**b**) and extracellular viral load (**c**) were detected by Western blotting and real-time RT-PCR, respectively. Brefeldin A inhibited IBDV growth both in normal cells (I) and overexpressed CSGalNAcT2 cells (III). CSGalNAcT2 enhanced IBDV propagation, because the mature VP2 levels and viral load were promoted by overexpression of CSGalNAcT2 (IV). However, this enhancement was inhibited by Brefeldin A (III), which suggested that the integrity of the Golgi structure is involved in the function of CSGalNAcT2.

Brefeldin A is one of the most thoroughly investigated Golgi-disturbing agents, which can destroy the Golgi apparatus structure [37]. Thus, Brefeldin A was used to assess whether the effect of CSGalNAcT2 overexpression on IBDV growth was dependent on the Golgi apparatus. The exogenous CSGalNAcT2 protein in DF1 cells became dispersed in the presence of Brefeldin A, but remained perinuclear in the absence of Brefeldin A treatment (Figure 6a), which suggested that Brefeldin A indeed dispersed the structure of the Golgi apparatus and that the exogenous CSGalNAcT2 was located in the Golgi apparatus. 

Firstly, we verified the effect of Brefeldin A on IBDV replication in the absence of CSGalNAcT2 overexpression. Both the content of intracellular mature VP2 and the genomic RNA copies of IBDV in the supernatant decreased during Brefeldin A treatment (Figure 6b,c), indicating that the integrity of the Golgi apparatus was involved in IBDV infection. 

Subsequently, we investigated whether enhancement of IBDV growth by CSGalNAcT2 overexpression could be inhibited by Brefeldin A treatment. After overexpression of CSGalNAcT2, both the mature VP2 levels and viral load were increased (Figure 6b,c), but this enhancing effect was inhibited by Brefeldin A treatment (see groups III and IV in Figure 6b,c). These results indicated that the effect of CSGalNAcT2 on IBDV was dependent on the integrity of the structure of the Golgi apparatus.

## 4. Discussion

To explore the cellular interactions associated with the multifunctional capsid protein VP2 of IBDV, we used a yeast two-hybrid approach, and identified CSGalNAcT2, located in Golgi apparatus, as a novel binding partner of VP2, and further confirmed the interaction using co-immunoprecipitation and confocal laser scanning microscopy assays. Analysis of differentially expressed genes showed that CSGalNAcT2 was up-regulated during IBDV infection; this was also confirmed by real-time RT-PCR. 

CSGalNAcT2 is a typical type II transmembrane protein located in the Golgi apparatus [38]. The Golgi apparatus is involved in the processing, modification, transportation, and secretion of different viral proteins [39,40]. In addition, many viruses, such as those belonging to the *Togaviridae* (a rubella virus) [41], *Bunyaviridae* (a Bunyamwera virus) [42], and *Arteriviridae* (a porcine reproductive and respiratory syndrome virus) [43], complete their assembly and form mature viral particles in the Golgi apparatus. However, to our knowledge, the role of CSGalNAcT2 in virus infection has not yet been reported. This prompted us to assess the influence of this Golgi protein on the life cycle of IBDV. We found that overexpression of CSGalNAcT2 could promote the growth of IBDV, while viral replication was decreased by the knockdown of the gene encoding CSGalNAcT2, thus verifying that the Golgi protein CSGalNAcT2 is beneficial to IBDV infection.

The Golgi apparatus is a dynamic structure that constantly exchanges proteins and lipids with other organelles. The protein processing and trafficking function of the Golgi is intimately linked to multiple intracellular signaling pathways [44]. To study whether the function of CSGalNAcT2 on IBDV is dependent on the Golgi apparatus, the Golgi-specific compound, Brefeldin A, was used to destroy the structure of the Golgi apparatus. After treatment with Brefeldin A, the content of viral proteins and genomic RNA copies of IBDV decreased, as compared to untreated controls. Even in cells overexpressing CSGalNAcT2, the promotion to IBDV replication were inhibited by this treatment. These results indicated that the function of CSGalNAcT2 was dependent on the integrity of the structure of the Golgi apparatus.

Recently, an imaging study showed that the three components of the IBDV ribonucleoprotein complex (VP1, VP3, and dsRNA) co-localized in the Golgi apparatus [45], which indicated that the Golgi apparatus is involved in the assembly of IBDV. As the capsid protein, the maturity and modification of VP2 is indispensable for the assembly of IBDV [13]. In this study, we found VP2 was distributed throughout the cytoplasm when independently expressed, while this distribution was markedly altered to accumulate around the cell nucleus upon co-expression with CSGalNAcT2 because the location of CSGalNAcT2 is closed to nucleus. So we suggested that pVP2 or VP2 could be recruited to the Golgi apparatus via its interaction with CSGalNAcT2. Ona *et al.* also reported that pVP2 co-localized with VP3 in the perinuclear area [46]. Along with our findings, these results indicated that the Golgi apparatus may be involved in IBDV assembly and infection. In addition, upon the interaction, the *N*-acetylgalactosaminyltransferase activity of CSGalNAcT2 might also take part in the glycosylated modification of VP2 and indirectly affected the replication of IBDV. It has been reported that the capsid protein VP2 of infectious pancreatic necrosis virus (IPNV), another member of the family *Birnaviridae* with a similar genome structure to IBDV, was an O-linked glycoprotein [47], which implied that the possible glycosylated modification of VP2 existed during IBDV infection. Moreover, it also could not rule out that the effect of CSGalNAcT2 on IBDV replication might be also associated with glycosylation level of some host proteins affected by CSGalNAcT2. Taken together, we therefore hypothesized that the up-regulation of CSGalNAcT2 after infection was required for recruiting more produced pVP2 or VP2 to the Golgi apparatus, thereby contributing to virus glycosylation or assembly, but the underlying molecular mechanism should be studied further in future. 

In conclusion, here, for the first time, we verified that CSGalNAcT2, located in the Golgi apparatus, interacted with VP2 and promoted the growth of IBDV. These results further demonstrated that the Golgi apparatus was indeed involved in the life cycle of IBDV, facilitating our understanding of this virus.
